# Emergency management of anaphylaxis and the impact of the new UK advanced life support guidelines

**DOI:** 10.1016/j.clinme.2025.100519

**Published:** 2025-09-30

**Authors:** Mahmoud Elshehawy, Madhavi Kadambi, Deborah Hughes, Daniel Clarke, Angela Cooper, Mohit Inani, Polat Goktas, Sarah Goddard, Lavanya Diwakar

**Affiliations:** a Acute Medicine, Shrewsbury and Telford Hospitals NHS Trust, Shrewsbury, United Kingdom; b Specialist Registrar in Allergy, Guy's and St. Thomas' NHS Foundation Trust, London, United Kingdom; c Department of Immunology and Allergy, UHNM, Stoke on Trent, United Kingdom; d Associate Professor in Computer Science, Sabanci University, Ankara, Turkey; e Honorary Associate Professor in Health Economics, University of Birmingham, Birmingham, United Kingdom

## Abstract

**Background:**

Anaphylaxis is a severe, potentially life-threatening allergic reaction that requires urgent and effective management. The UK Resuscitation Council updated its advanced life support (ALS) guidelines for anaphylaxis in 2021, emphasising early and repeated adrenaline administration, intravenous (IV) fluid use, and reduced reliance on antihistamines and steroids.

**Methods:**

A retrospective audit was carried out to compare the management of anaphylaxis at two English NHS hospitals, namely the University Hospital of North Midlands (UHNM) and the Shrewsbury and Telford Hospital (SATH), before (2018) and after (2022/23) the ALS guideline implementation. Adherence to NICE anaphylaxis guidance was also assessed.

**Results:**

Data from 272 patients revealed significant improvements in recognition of anaphylaxis in 2022 compared with 2018 (70.8% vs. 50%; *p*=0.001). The use of adrenaline and IV fluids increased, whereas the use of antihistamines and steroids declined, aligning with the new guidance. Tryptase measurement (checked in 45% of patients) and specialist referral rates (67% at UHNM vs. 3% at SATH; *p*=0.0001) remained suboptimal at both centres. A case example highlights the risks of misdiagnosis and adrenaline overuse in patients with recurrent urticarial presentations.

**Conclusion:**

Anaphylaxis management in these centres has changed in keeping with the new ALS guidelines, although antihistamines and steroids were still used in the acute management of around 50% of the patients. Adrenaline overuse may be an unintended consequence of the guideline, which needs monitoring. There may have been some improvement in anaphylaxis recognition, but serum tryptase measurement and referral to allergy specialists remain poor.

## Introduction

Anaphylaxis, though relatively rare, remains a serious and life-threatening condition. Prompt and effective management, particularly through early adrenaline administration, plays a critical role in improving patient outcomes (Simons *et al*).^4^

In May 2021, the Resuscitation Council UK (RCUK) updated its advanced life support (ALS) guidelines for anaphylaxis, marking a major change from the 2008 protocol. The new guidance emphasises early and repeated use of adrenaline, along with IV crystalloid boluses, for patients with refractory anaphylaxis (defined as those whose symptoms persist after two appropriate doses of adrenaline).^1^ Antihistamines, whose efficacy is probably limited to relieving cutaneous symptoms only,^2^ are now regarded as a third-line treatment. Steroids are no longer recommended for acute anaphylaxis management since their effectiveness is doubtful and they could be harmful in children.^2^ While concerns have been raised about overdiagnosis of anaphylaxis during the COVID-19 pandemic,^3^ there is a lack of data examining this issue outside of the pandemic context.

Aims were to audit the acute management of anaphylaxis in two large accident and emergency (A&E) departments, benchmarking practice against guidelines from the World Allergy Organization (WAO)^4^ and the National Institute for Health and Care Excellence (NICE).^5^ Data from 2018 were compared with those from 2022/23 to assess the impact of the updated RCUK guidance on anaphylaxis management at these centres.

## Methods

### Context

We conducted an audit to compare diagnosis and management of all patients presenting with anaphylaxis to the A&E departments at two large English hospitals between Jan–Dec 2018 (before the new ALS guidance) and Jan 2022 – Apr 2023 (after the new guidance was implemented). The University Hospital of North Midlands (UHNM) is a university hospital trust with a specialist allergy service that takes referrals from all regional hospitals, including Shrewsbury and Telford Hospitals NHS Trust (SATH). SATH includes two large district general hospitals which do not have an in-house allergy service. Together, these hospitals provide almost all emergency care for Staffordshire and Shropshire regions in the UK, serving approximately 1.6 million individuals.

### Population

Patients were identified using relevant ICD-10 codes (see supplement S1). Details of individual patient presentations, admissions and management were obtained from West Midlands Ambulance Service (WMAS) records and emergency department entries at both hospitals.

### Audit criteria

#### Anaphylaxis diagnosis

All patients coded as having anaphylaxis were reclassified by the investigators as per the WAO criteria. Anaphylaxis was defined as the sudden onset of two or more of the following symptoms:

• Skin: generalised rash, hives, itching, swollen lips/tongue/uvula.
• Respiratory: shortness of breath, wheeze, cough, stridor, hypoxaemia.
• Cardiovascular or end organ dysfunction: hypotension, hypotonia, incontinence.
• Gastrointestinal: crampy abdominal pain, diarrhoea.

### Management of anaphylaxis

• Administration of adrenaline, IV fluids, antihistamines and steroids as per the relevant ALS guidance.
• Timed serum tryptase samples (one soon after and another within 1–2 hours of presentation at A&E)^5^ (Normal level: 1–15 ng/mL).
• Referral to specialist allergy services upon discharge.^5^

#### Analysis

Statistical analysis was performed using Stata 18 and Microsoft Excel 2013. The respective hospital audit departments approved the audit.^5^

## Results

### Patient distribution and demographics

A total of 272 patients reviewed in the A&E departments of both hospitals were included in this audit. The demographic data and other characteristics are summarised in Table 1.

**Table 1:** Summary of patient characteristics.

Characteristic	2018 (n=100)	2022/23 (n=172)
Centre UHNM	65 (65%)	98 (57%)
Age (mean±SD)	39.33±20.49	40.03±22.85
Age range	0–91	1–90
Paediatric (≤18 yrs)	11 (11%)	34 (20%)
Male	40 (40%)	79 (45.9%)
Brought in by ambulance	71 (71%)	114 (66.3%)
Repeat attendances	4 (4%)	7 (4.1%)

### Anaphylaxis diagnosis

**Fig. 1:**
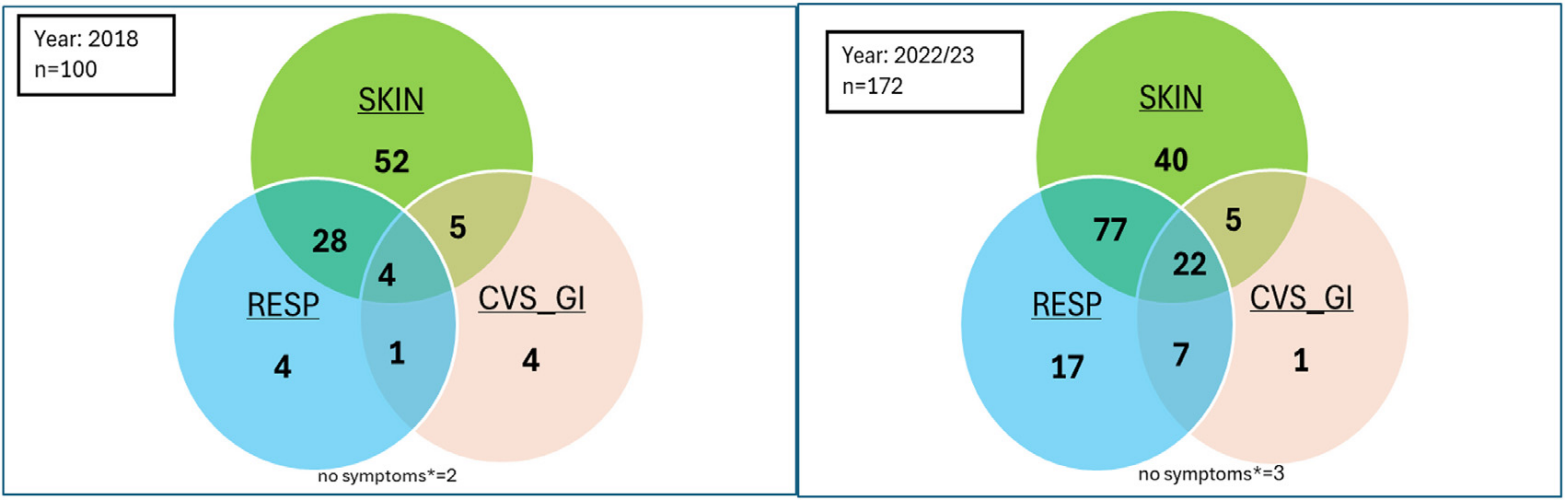
Venn diagram showing the clinical features of patients presenting with anaphylaxis. Those presenting with cardiovascular and/or gastrointestinal symptoms are shown within a composite category (CVS-GI).

Fig. 1 shows the presenting symptoms of the patients. More patients were labelled accurately as per WAO criteria as having anaphylaxis in 2022/23 compared with 2018 (Table 2). However, over half the patients in 2018 and about a quarter (23%) in 2022/23 were coded as having anaphylaxis despite only presenting with cutaneous symptoms.

**Table 2:** Summary of diagnosis and management.

Characteristic	2018 (n=100)	2022/23 (n=172)	comment
Satisfies WAO criteria for diagnosis	50 (50%)	121 (70.8%)	*p*=0.001
Refractory anaphylaxis^a^	8 (8%)	25 (14.5%)	*p*=0.111
Tryptase checked	29 (29%)	92 (53.5%)	*p*=0.0001
Admitted	63 (63%)	70 (40.7%)	*p*=0.0001
Referred to specialist clinic (all patients)	37 (37%)	64 (37.2%)	
Referred to specialist (UHNM)	32 (49.2%)	62 (63.3%)	*p*=0.06
Referred to specialist (SATH)	5 (14.3%)	2 (2.7%)	*p*=0.06

a Refers to patients receiving >2 doses of adrenaline.

### Management of anaphylaxis

**Fig. 2:**
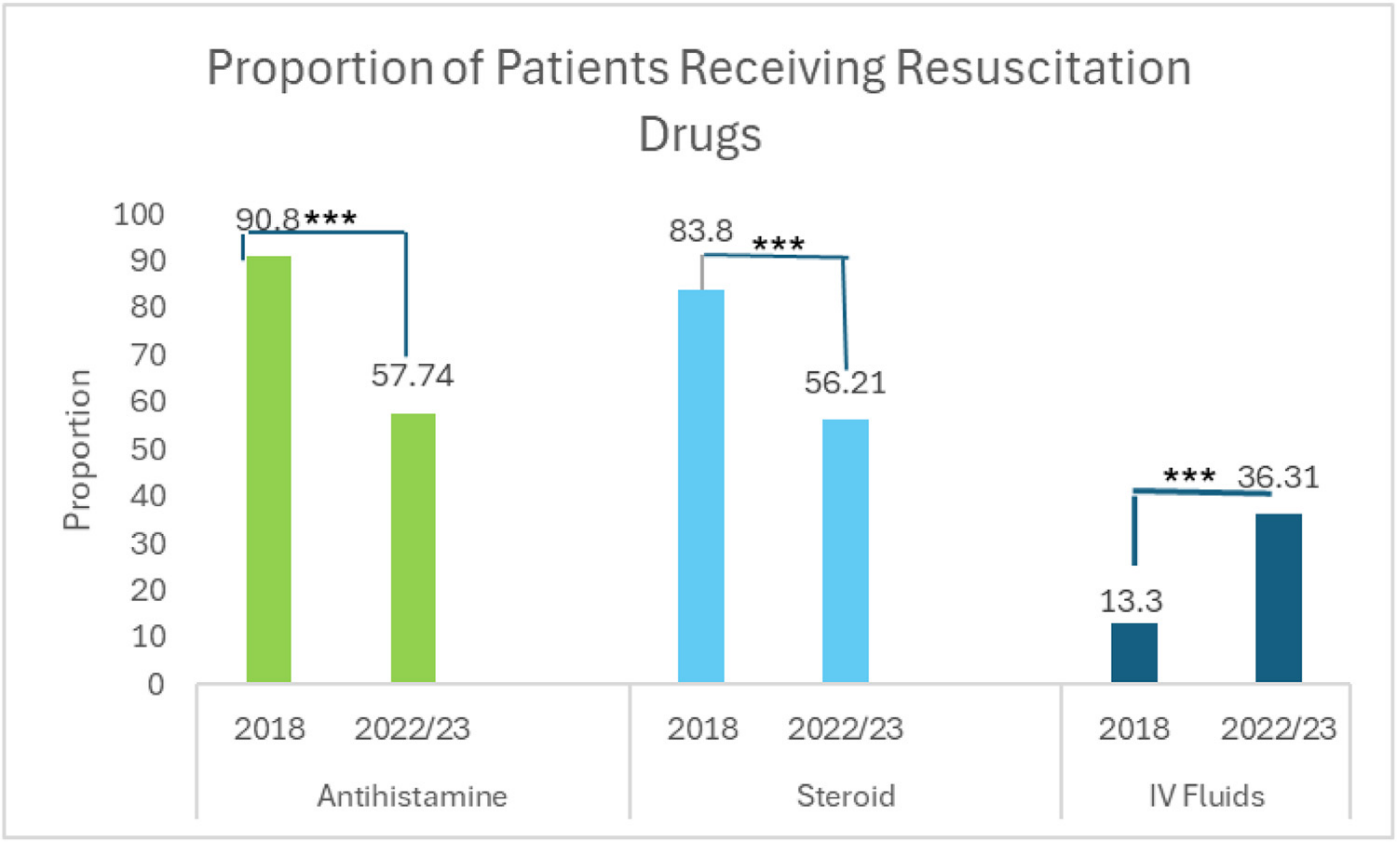
There was an increase in the use of IV fluids and decrease in steroid and antihistamine use as a direct result of the new guidelines.

There was a significant decrease in antihistamine and steroid use, along with a significant increase in IV fluid administration, reflecting the updated guidelines (Fig. 2).

The number of individuals managed as having refractory anaphylaxis increased in 2022/23, although this was not statistically significant. Table 2 summarises the changes in management and outcomes in between the 2 cohorts of patients. A detailed breakdown of the total number of adrenaline doses given per episode is shown in Table 3.

**Table 3:** Total adrenaline doses administered per episode in each cohort.

Total adrenaline doses	2018 (n, %)	2022/23 (n, %)
0	14 (14%)	47 (27.3%)
1-2	78 (78%)	100 (58.14%)
3	6 (6%)	12 (6.9%)
4	2 (2%)	6 (3.5%)
5	0	5 (2.9%)
≥6	0	2 (1.2%)

Tryptase measurement improved slightly in 2022/23, although nearly half of the patients still did not have a sample sent from emergency departments. Patients whose presenting symptoms met the WAO criteria were more likely to have their tryptase measured (*p*=0.016). In our study, there was no significant correlation between meeting WAO criteria and raised tryptase levels (*p*=0.57).

### Adrenaline use

The number of individuals receiving higher doses of adrenaline (>2) increased between 2018 and 2022/23 (*p*=0.111). Whereas no one received more than four doses of adrenaline in 2018, a few patients had higher doses in 2022/23. In 2022/23, significantly more patients with refractory anaphylaxis were treated with steroids and IV fluids (see table S1 and case description in supplement).

A considerable number of individuals (n=61; 22.4%) with a diagnosis of anaphylaxis did not receive adrenaline. Around half of these individuals (n=31; 50.8%) satisfied WAO criteria for the diagnosis of anaphylaxis (see Table S2 and Fig. S1).

### Clinical management and outcomes

There were no deaths reported due to anaphylaxis at either hospital during the study period. Admission rates for patients with anaphylaxis fell in both hospitals in 2022/23. 67% and under 3% of patients presenting to UHNM and SATH respectively with anaphylaxis in 2022/23 were referred to specialist clinics (*p*=0.0001).

## Discussion

This audit aimed to ascertain current practices with regards to diagnosis and emergency management of anaphylaxis in the Staffordshire and Shropshire region of the UK, before and after the implementation of new ALS guidelines. We found that adrenaline and IV fluid use has notably increased during immediate resuscitation, and the administration of antihistamines and steroids has decreased, in keeping with the new guidance. There was a significant improvement in the recognition of anaphylaxis by A&E and paramedical staff, although about 30% of patients still did not meet WAO criteria in 2022/23. A fifth of patients, half of whom met the WAO criteria for anaphylaxis, did not receive any adrenaline. Tryptase measurement in A&E remains poor, although there was a significant improvement in 2022/23. Only 3% of patients from SATH and 65% of patients from UHNM were referred to specialist clinics.

Patient outcomes were stable – there were no deaths due to anaphylaxis during the study period, and the number of hospital admissions has significantly decreased. However, the latter finding may be related to changes in hospital admission policies post-pandemic rather than to the new resuscitation protocols.

Some patients received very high doses of adrenaline, which, in some cases, could be related to a misinterpretation of the severity of the clinical presentation. There is concern regarding the overuse of adrenaline in some patients with symptoms that, under previous guidance, might have been managed with antihistamines. For instance, the case example provided in the Supplementary Material illustrates how recurrent urticarial episodes were initially misdiagnosed as anaphylaxis, leading to excessive adrenaline administration and associated complications.

The importance of adrenaline in the early management of anaphylaxis symptoms is firmly established. A UK-based study showed that delaying the administration of adrenaline can be detrimental to anaphylaxis management.^6^ The reasons for the removal of antihistamines as a first-line treatment in the current guideline are not entirely clear. A Canadian study suggested that early antihistamine use in severe allergic reactions may reduce the progression of anaphylaxis.^7^ A third of all patients in our study had skin symptoms only and could have been better managed with antihistamines than adrenaline.

Our audit is the first to study the effect of the new ALS guidance on the acute management of anaphylaxis. Our data were obtained from ambulance and A&E entries and, therefore, provide a comprehensive overview of the presentation and management of the included patients. We compared a centre with an in-house allergy department and one without, highlighting the heterogeneity in care and referral patterns for the patients.

Since this is a retrospective study, it is possible that some crucial clues in patient presentation and management were missed in the documentation. In addition, a direct comparison ignores any intrinsic differences between the cohorts, which may contribute to the variation in management. Administration of other relevant drugs such as oxygen was not explored in this audit. We did not include patients who had anaphylaxis in the wards or operating theatres in either of the hospitals.

Data from other centres may help improve understanding of the impact of new guidelines and their impact on patient outcomes. These data will be presented to the clinical staff at both hospitals via grand round presentations and A&E departmental teaching sessions. We will reaudit the data in due course.

## Conclusion

There has been a significant change in the acute management of anaphylaxis following the implementation of the 2021 ALS guidelines. There has been an improvement in the recognition of anaphylaxis, although a considerable number of patients are likely misdiagnosed. There was some concern regarding the over-utilisation of adrenaline in our cohort, but this was not statistically significant. This should be monitored in future studies. Most patients were not referred to allergy specialist clinics, and a considerable number do not have tryptase measured in A&E. We have identified specific areas that need to be addressed in our region to improve the management of these patients.

## Ethical approval and consent to participate

This study used retrospectively collected, anonymised data and did not involve patient contact or intervention. Individual consent was not needed. In accordance with UK Health Research Authority (HRA) guidance, Research Ethics Committee approval was not required. Local institutional governance procedures were followed.

## Consent for publication

Written informed patient consent was obtained for case presentation in the supplement section.

## Funding

This study did not receive any specific funding. LD currently holds an NIHR development and skills enhancement fellowship (NIHR303772). The views expressed in this article are those of the author and not necessarily those of the NIHR or the Department of Health and Social Care.

## Data availability statement

The data that support the findings of this study are available from the corresponding author upon reasonable request.

## CRediT authorship contribution statement

**Mahmoud Elshehawy:** Writing – original draft, Methodology, Investigation. **Madhavi Kadambi:** Writing – review & editing, Resources, Project administration, Investigation. **Deborah Hughes:** Writing – review & editing, Resources, Investigation. **Daniel Clarke:** Investigation, Writing – review & editing. **Angela Cooper:** Writing – review & editing, Investigation. **Mohit Inani:** Writing – review & editing, Investigation. **Polat Goktas:** Writing – review & editing, Visualization. **Sarah Goddard:** Writing – review & editing, Methodology, Investigation. **Lavanya Diwakar:** Writing – original draft, Supervision, Methodology, Investigation, Formal analysis, Conceptualization.

## Declaration of competing interest

The authors declare that they have no known competing financial interests or personal relationships that could have appeared to influence the work reported in this paper.

## Appendix Supplementary materials

Image, application 1
