# Supplementary material for: Emergency management of anaphylaxis and the impact of the new UK advanced life support guidelines
Source: Clin Med (Lond). 2025 Sep 30;25(6):100519. doi: 10.1016/j.clinme.2025.100519 (PMC12581685; doi:10.1016/j.clinme.2025.100519)
Supplement: Supplementary file 1 [file mmc1.docx]

**Supplementary Material: Detailed Case History**

A 29-year-old primary school teacher called 999 in April 2024 with a history of wheeze and noisy breathing following a bee sting. She is known to have asthma, mixed anxiety depression disorder, and mild psoriasis. Initial observations showed a heart rate of 123 bpm, BP of 119/67 mmHg, and oxygen saturation of 100% on air. GCS was 15/15. She described having hives around the area of the sting.

She self-administered two Epipens and was treated by paramedics with four doses of adrenaline and two salbutamol nebulisers. In A&E, she received IV boluses of adrenaline due to reduced blood pressure, which was later attributed to myocardial stunning caused by excessive adrenaline administration. The infusion stopped after a couple of hours, and she made a full recovery. Tryptase levels measured at 2 and 3 hours post-incident were 4.4 and 4.5 mcg/L, respectively.

Further review in the allergy clinic revealed that she had experienced other similar episodes of wheeze and a mildly swollen tongue in June 2023 and August 2022, which were treated by paramedics with salbutamol nebulisers and four doses of adrenaline each (in addition to the two Epipens she self-administered).Baseline observations were normal on both occasions as was the serum tryptase.

She also presented to A&E in Nov 2019 after waking up with a rash and shortness of breath which responded to oral antihistamines and steroids.

Investigations were negative for bee and wasp allergy (Specific IgE grade 0).

She was diagnosed with possible chronic spontaneous urticaria and angioedema and advised to take regular antihistamine in the first instance. She was provided with an emergency management plan which was shared with the A&E department advising that adrenaline not be used in management of urticaria and that antihistamines and salbutamol (where indicated) should be the treatment of choice unless there was a documented fall in blood pressure and/or oxygen saturation.

She was last reviewed in the allergy unit 6 months ago and reports being well with no recent episodes of urticaria.

Table S1: Management of refractory anaphylaxis in the Emergency department

|  | **2018** | | | **2022/23** | | |
| --- | --- | --- | --- | --- | --- | --- |
|  | Responded to Adrenaline (n=92) | Refractory (n=8) | P value | Responded to Adrenaline (n=147) | Refractory (n=25) | P value |
| Meet WAO criteria | 45 (48.9%) | 5 (62.5%) | 0.461 | 101 (58.7%) | 21 (84%) | 0.120 |
| Antihistamine admininstered | 82 (89.1%) | 7 (87.5%) | 0.735 | 81 (47.1%) | 16 (64%) | 0.339 |
| Steroid administered | 77 (83.7%) | 6 (75%) | 0.479 | 75 (51%) | 20 (80%) | 0.009 |
| IV fluids given | 10 (10.9%) | 3 (37.5%) | 0.017 | 45 (30.6%) | 16 (64%) | 0.001 |

Table S2: Characteristics of individuals who were not given adrenaline for anaphylaxis (n=61)

| Characteristic | Number (%) |
| --- | --- |
| Children (≤18yrs) | 14 (23) |
| Meet WAO criteria | 31 (50.8) |
| Tryptase not checked | 37 (60.7) |
| Tryptase normal* | 12 (19.7) |
| Tryptase raised* | 12 (19.7) |

*serum tryptase normal range:1-14 ng/ml; an elevation of more than 20%+2ng/ml over baseline was also considered as significant

Figure S2: Clinical features of patients who did not receive adrenaline (n=61)*

| 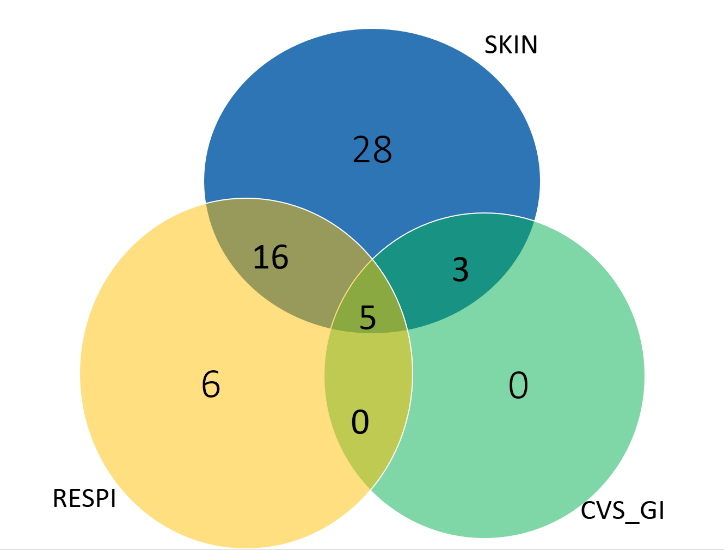 |
| --- |

*3 patients had no skin/ respiratory/ GI or cardiovascular symptoms
